# Corrigendum to: Off‐target inhibition by active site‐targeting SHP2 inhibitors

**DOI:** 10.1002/2211-5463.12754

**Published:** 2019-12-02

**Authors:** Ryouhei Tsutsumi, Hao Ran, Jörg Rademann, Benjamin G. Neel

**Affiliations:** ^1^ Laura and Isaac Perlmutter Cancer Center NYU Langone Health New York NY USA; ^2^ Institute of Pharmacy, Pharmaceutical and Medicinal Chemistry Freie Universität Berlin Königin‐Luise‐Str. 2+4 14195 Berlin Germany

Correspondence

B. G. Neel, Laura and Isaac Perlmutter, Cancer Center, NYU Langone Health, New York, NY, USA

E‐mail: benjamin.neel@nyulangone.org

The list of authors of the paper by Tsutsumi *et al*. 1 was incomplete. The full list of authors and their contributions are given here.

## Author contributions

RT and BGN conceived and designed the experiments and wrote the manuscript. RT and HR performed the experiments. JR synthesized one of the inhibitors. BGN supervised the research.
